# The architecture of embodied cue integration: insight from the “motivation as cognition” perspective

**DOI:** 10.3389/fpsyg.2015.00658

**Published:** 2015-05-21

**Authors:** Idit Shalev

**Affiliations:** Department of Education and the Zlotowski Center of Neuroscience, Ben-Gurion University of the NegevBeer Sheva, Israel

**Keywords:** concept activation, cue integration, embodied cognition, feature integration, goal systems theory, motivation as cognition, social cognition

## The malleable nature of embodied cues in judgment and behavior

At the core of embodied cognition research is the assumption that higher level processing is grounded in the organism's lower level sensory and motor experiences (Barsalou, 1999, 2008; Meier et al., 2012; Winkielman et al., 2015b). Past research of perceptual multimodal cue integration has demonstrated that several mechanisms underlie perceptual integration (Treisman and Gelade, 1980; Zmigrod and Hommel, 2013). Based on embodied cognition theory, which indicates that activation automatically spreads from concepts driven by experiences in the physical world to their metaphorically-related social concepts (for reviews, Williams et al., 2009; Meier et al., 2012), it was proposed that to produce action, embodied cues associate between lower level and higher level cues. However, little is known about the factors that modulate this integration. This gap in the literature is of relevance because research of embodied cognition has demonstrated that perceptual symbols can lead to different patterns of activation across different contexts (Barsalou, 2008), which makes predictions about judgment and behavior difficult. For example, the associations between physical warmth/coldness and psychological warmth/coldness across different contexts yielded both assimilative effects (e.g., physical warmth increases psychological warmth) (Williams and Bargh, 2008) and contrast effects (e.g., physical coldness increases the need for social warmth) (Zhong and Leonardelli, 2008; Bargh and Shalev, 2012; Shalev and Bargh, 2014; Zhang and Risen, 2014).

Following the recent pragmatic turn in cognitive science, according to which cognitive processes and their underlying neural activity patterns should be studied primarily with respect to their roles in action generation (Glenberg et al., 2013), I argue that embodied cues are integrated according to their momentary functions within each individual's system of goals. Conceptualized as cognitive representations of desired end-points that affect evaluations, emotions and behaviors (Fishbach and Ferguson, 2007), goals serve as reference points toward which behavior is directed. I suggest that analyzing embodied cue integration from the “motivation as cognition” perspective (Kruglanski et al., 2002) may add to our understanding of which cues are perceived, what response is determined as appropriate in a given situation, and why different judgments and behaviors may be elicited by the activation of similar sets of embodied cues. In the sections below, I will discuss three types of constraints that stem from the “motivation as cognition” perspective, including the motivational properties of embodied cue integration (Eitam and Higgins, 2010), the allocational properties of embodied cue integration (based on attentional resource-limitation, see Kahneman, 1973), and the structural properties of cognitive-interconnectedness and uniqueness (Kruglanski et al., 2002). The three types of constraints, adopted from goal systems theory (Kruglanski et al., 2002), were invoked to explain the process of embodied cue integration.

## What is the “motivation as cognition” perspective?

The “motivation as cognition” perspective assigned distinct functions to motivational and cognitive variables. A basic assumption is that motivation can fluctuate from one moment to the next, thus determining the extent to which any kind of information (strategic and peripheral; conscious and unconscious) is processed (Kruglanski and Thompson, 1999). Mental representations of motivational networks comprise inter-connected goals and means that may be automatically activated simultaneously by different cues, and as such, they may compete with each other for mental resources (Kruglanski et al., 2002). Likewise, according to this approach, several cognitive properties set the constraints within which the motivational properties may express themselves. Because both motivation and embodied cognition are types of cognition, this set of cognitive constraints may explain the way motivation influences embodied cue integration. In the sections below, I will discuss the constraints on cue integration, including the motivational properties of embodied cue integration (Eitam and Higgins, 2010), the allocational properties of embodied cue integration (based on attentional resource-limitation, see Kahneman, 1973), and the structural properties of cognitive-interconnectedness and uniqueness (Kruglanski et al., 2002).

## The motivational properties of embodied cue integration

The first assumption of embodied cue integration is that because numerous sensori-motor cues can serve as the material for multiple social inferences, a selection process is needed to determine which cues to integrate in a given situation to create meaning. I suggest that perceptual or conceptual saliency depends on whether a mental representation reflects the individual's momentary goals, and what, if any, relationship those goals have with the salient cues in the immediate environment of the individual (Balcetis and Dunning, 2009; Balcetis et al., 2012). A similar line of thought was suggested by De Houwer (2009), indicating that associative learning effects are determined not only by the direct experience of events but also by prior knowledge and instructions. Pursuing this logic, Eitam and Higgins (2010) suggest that whether, and the degree to which, a stimulated mental representation is activated reflects the relative weights of one or any combination of three sources of motivational relevance: value relevance, or the extent to which acting on a mental representation will bring about desired results and/or prevent undesired results; control relevance, or the efficacy with which the activated representation makes things happen; and truth relevance, which establishes what is real. Thus, the relative extents to which these sources are relevant to the individual's needs determines the level and duration of activation, regardless of the content of the representation.

Indeed, recent findings of embodied cognition research have provided strong evidence for the effect of motivational relevance on cue integration. For example, one study demonstrated the source of value relevance by showing that the adoption of approach-type postures (e.g., leaning forward) was associated with increases in neural activation characteristic of approach situations (Harmon-Jones et al., 2011). In another study, the performance of avoidance type movements (pushing a shopping cart as opposed to holding it) was associated with fewer reward-oriented consumer choices at the checkout counter (Van den Bergh et al., 2011). The source of control relevance was demonstrated by showing that embodied simulations of facial expressions were expressed for conceptual understanding only if they were relevant to solving the task at hand (Niedenthal et al., 2009), indicating that embodiments are not passive byproducts of conceptual processing (Winkielman et al., 2015a). Finally, the source of truth relevance was shown in a study where participants were asked to verify or deny that a certain object has a certain property (i.e., answer a question such as “Do cats have wings?”). The results showed that the speed of property verification was related to the perceptual salience of the feature in question (Solomon and Barsalou, 2004). For example, property verification was quicker the more conspicuous the property, presumably because such properties are easier to see in a recalled or simulated visual representation.

## The allocation properties of embodied cue integration

The second assumption of embodied cue integration is that the fundamental allocation property relies on limited mental resources. From that perspective, the allocation of cognitive resources has a functional purpose, namely, to minimize the extent to which mental resources are exploited in the creation of unified percepts. The property of limited resources is demonstrated, for instance, by motor fluency effects observed only when individuals are involved in monitoring situational constraints. For example, research showed that compared with rigid right-handers, flexible right-handers recalled product orientations better and showed a preference for objects on which the handle was oriented in the direction of the hand used for grasping (Eelen et al., 2013).

Another application for the limited resources effect is demonstrated by the switching cost entailed in shifting attention from one modality to another (e.g., from audition to vision), indicating that the second stimulus is processed more slowly than it would have been had the two stimuli used the same modality (e.g., Spence et al., 2001). The switching cost was also demonstrated by the perceptual simulation approach, indicating that verifying the properties of concepts in the auditory modality was slower after verifying a property in a different modality than after verifying one in the same modality (Pecher et al., 2003).

The limited resources assumption has several consequences. First, I suggest that the integration process is fundamentally economic and that it operates automatically by activating samples of the interconnection between sounds, sights, and other sensory signals that were encoded in memory based on previous learning (Brunel et al., 2009; Zmigrod et al., 2009; Bargh and Morsella, 2010; Vallet et al., 2010). Evidence for automatic activation is based on the ideomotor theory, which assumes the existence of an automatic action–effect integration mechanism that binds motor patterns and action effect representation (Chartrand and Bargh, 1999; Zmigrod and Hommel, 2013). Second, as was recently proposed by Winkielman et al. (2015b), I argue that non conscious automatic signals, including fluency and a sense of coherence, inform fundamental cognitive and social judgments (Winkielman and Schooler, 2011; Schwarz, 2015), thereby consuming fewer cognitive resources.

## The structural properties of embodied cue integration

The third assumption of embodied cue integration is that unified percept configurations are influenced by sensori-motor cue interconnections, including the form and associative strength of those interconnections. The strength of association between multi-modal units is positively related to the uniqueness of the interconnections (Kruglanski et al., 2002).

This dynamic helps explain why specific embodied metaphors have stronger associative links than other metaphors with sensori-motor cues. A possible explanation could be that the repetition of specific social inferences across different contexts in response to sensori-motor contextual cues occurs when the strength of the association is high or in populations where this motivation is accessible. For example, evidence that washing one's hands also “washes away” feelings of guilt was found not only in a normal population (Zhong and Liljenquist, 2006; Lee and Schwarz, 2010, 2011), but also among patients with obsessive compulsive disorder in whom the association between contents related to physical and psychological cleanliness is stronger (Reuven et al., 2013). Likewise, research indicates that core metaphors (e.g., temperature, distance) are associated with multiple conceptual phrases (Schnall, 2014), suggesting possible variability in the strengths of the associations between sub-metaphors associated with the core metaphor. Likewise, individual and cultural differences also influence these associative strengths and may have an impact on the replicability of findings (Shalev and Bargh, 2014).

Another structural application of embodied cue integration is the substitutability relations of cues associated with an identical mental representation. For example, studies of the metaphorical links between physical and social temperatures (e.g., “warm smile,” “cold as ice”) showed that participants perceive others as “warmer” after they have held a warm rather than a cold cup of coffee (Williams and Bargh, 2008; IJzerman and Semin, 2009, 2010; Shalev and Bargh, 2011; Bargh and Shalev, 2012). Likewise, they experience a room as physically colder after having been socially rejected (Zhong and Leonardelli, 2008), indicating a possible substitutability between physical and semantic psychological concepts.

## Conclusions

This paper suggests that several constraints based on the “motivation as cognition” paradigm modulate the interrelations between perception, emotion and action, and in so doing, they influence embodied cue integration in both bottom-up and top-down manners. On the one hand, active goals influence the feasibility of relevant embodied cues. On the other hand, the perceiver's likelihood of drawing a specific inference may be proportional to the strengths of the associations between contextual cues and sights and sounds encountered by the individual (Zaki, 2013). Based on this reasoning, I suggest that inferences are highly flexible and context-dependent, and therefore, they vary in accordance with situational framing effects (Loersch and Payne, 2011; Wiltshire et al., 2015). As with other psychological phenomena, individual differences (e.g., physical disability, mental health conditions) could increase the likelihood that specific motivational states will be associated with particular embodied cues. Likewise, the repetition of specific social inferences in response to similar sensori-motor contextual cues is possible and may depend on the strength of the association within unique cue configurations. The contribution of the embodied cue integration approach goes beyond explaining the variability of findings across different contexts. By combining cognitive architecture, semantic metaphoric configurations and structural motivational properties, embodied cue integration offers a possible path for integrating different lines of thought in the field of embodied cognition.

### Conflict of interest statement

The author declares that the research was conducted in the absence of any commercial or financial relationships that could be construed as a potential conflict of interest.
